# Shared intentional engagement through language and phenomenal experience

**DOI:** 10.3389/fpsyg.2014.01016

**Published:** 2014-10-08

**Authors:** Christoph Durt

**Affiliations:** University of Heidelberg, Phenomenological Section, Clinic for General PsychiatryHeidelberg, Germany

**Keywords:** interaction, engagement, intentionality, consciousness, phenomenology, phenomenal experience, experience, sensations

## Abstract

This article introduces the notion of shared intentional engagement and argues that the current debate around intersubjective interaction can profit from taking that notion into account. Shared intentional engagement holds between people when they relate together to the same meaningful entities. For instance, when people talk about something, they share intentional engagement as long as they don't talk past each other. But what if the entity talked about involves perceptual experience—is the quality of one's experiences not something that cannot be conveyed to others through language? Against this widespread idea, this article takes up philosophical arguments for the intersubjectivity of, on the one hand, language, and, on the other hand, phenomenal experience. It contents that language and phenomenal experience both exhibit shared structures that enable shared intentional engagement. It then considers an example for how this result matches well with empirical research on “pop out” experiences. Because shared intentional engagement is fundamental for all kinds of human interaction, it necessitates interdisciplinary investigations that are frequently hindered by the assumption that the phenomenal experiences of humans are hidden to others.

Intersubjective interaction is becoming an increasingly important topic in the literature on cognitive science, for good reason. Intersubjective interaction is a pervasive feature of human life, and thinking about it is apt to show and potentially overcome the limits of the standard inferential approach to other minds. This article looks into several recent attempts to do so, and contents that the notion of shared intentional engagement can contribute to a better understanding of intersubjective interaction. It considers the role of language and phenomenal experience for intersubjective interaction, and argues that both provide the structures that enable shared intentional engagement.

An example for the inferential approach to other minds is “theory theory,” according to which the participating subjects apply their own and possibly implicit theories about the “mental states” of others by means of a folk psychology, which is then either falsified or confirmed in the interaction. Another example is “simulation theory,” according to which one does not need a theory of the “mental states” of others, but rather employs one's “own mind as a model, with which we simulate—create ‘as if’ or pretend beliefs, desires, intentional states—and then project these mental states into the mind of the other person to explain or predict their behavior” (Gallagher, 2009, p. 290). Theory theory assumes that knowledge about the mental states of others is reached through a theory of their behavior. Simulation theory contents that this is done by relating them to one's own states of mind, maybe through physiological mechanisms like those manifested in “mirror neurons.”

The inferential approach attempts to explain intersubjective interaction through an observation based model. Observation surely is important for intersubjective interaction. Yet, it is a one-way relation: the observer is observing the actor, but the actor may not even know of the observation. Intersubjective interaction, in contrast, is never just a one-way relation. There are a number of recent attempts to understand what characterizes intersubjective interaction, such as the distinction between engagement and coupling by De Jaegher et al. (2010). “Coupling” refers to exchanges that could be had between lifeless bodies, such as the exchange of heat. Engagement, in contrast, is the “qualitative aspect of social interaction as it starts to ‘take over’ and acquires a momentum of its own” (p. 441). Other authors, such as Schilbach et al., point out that social cognition does not happen between detached observers, and contend that there often is “emotional engagement” (Schilbach et al., 2013, p. 396).

While the details of the proposals of these authors are quite different, they all make an important observation: coupling is not enough for intersubjective interaction, there also has to be engagement. The notion of engagement connects to that of the second-person approach, according to which “recognizing and being recognized by a *You* is primary for understanding other people” (Reddy, 2008, p. 233). Part of what engagement means is that the actors recognize each other. Engaged interaction is a second-person relation in that the other is recognized as an interactor. That means that she or he is recognized as somebody who does not only act, but also reacts to the other's actions, who asks and responds, who has expectations, and who enters into obligations through her or his actions. Each action allows some and forbids other future actions, which is a reason for why engagement has a “momentum of its own.” Because such interactions are likely to involve emotion, emotional engagement is an important form of engagement.

In this article, I would like to draw attention to another form of engagement that is of fundamental importance for all human interaction. It may be dubbed *shared intentional engagement*. Shared intentional engagement is the engagement people are in when they relate together to an action, belief, idea, symbol, object, or other meaningful entity. For instance, when two people talk about an entity, they share intentional engagement. Of course, people also talk past each other. When that happens, they cease to engage in shared intentionality with regard to the meaning of their speech. That does not mean that in shared intentional engagement each subject has exactly the same understanding of the entities intended. Nor is the meaning of the intended entities up to the individuals; each actor can learn new things about the entity. Also, shared intentional engagement does not need to be part of a full-blown language; it can be mediated through a language or not. It can consist in pre-linguistic and simple linguistic activities, such as when children and their parents relate to an object in “joint attention” (cf. Tomasello, 1999)—which does not have to mean that either interactor needs to have a representation of that object in her or his mind (cf. Reddy, 2008, p. 86). In this article, “language” is used in a wide sense. It is not restricted to representations, and it is thought to be intertwined with pre-linguistic behavior, which the interactors may or may not be able to verbalize.

Language and phenomenal experience are often thought to be the two constituents of a dichotomy: On the one side, language is thought of as structuring otherwise unstructured phenomenal experience, which in itself only provides raw material. For instance, what pain and colors are, is thought to be due to the conventions of each language.^1^ Phenomenal experience, in contrast, is thought to be independent of language. For instance, the sensations one has when perceiving a color are thought to have a quality that is merely named in language. Studies such as that on joint attention would then show that shared intentional engagement can be had before and without language. But this is by no means the only interpretation. Such studies may also show that, on the one hand, language itself is rooted in human behavior, and, on the other, that pre-linguistic forms of shared intentional engagement are for normal speakers of a language shaped by that language. This paper argues that language and phenomenal experience both come together in shared intentional engagement.

Usually, shared intentionality is discussed under the heading of “collective intentionality.” Collective intentionality mainly concerns intentions that obviously cannot be had by one individual alone, such as the task of carrying an object that is too heavy for one person. A paradigmatic question in the discussions of collective intentionality is if “we-intentions” can be reduced to a sum of “I-intentions” (cf. Tuomela and Miller, 1988; Schmitz et al., 2013). The notion of shared intentional engagement, in contrast, is meant to draw attention to shared engagement in actions or entities that are typically done or had by only one individual. For instance, when a person talks about some pain she is feeling, she intends a pain that only she is having. That seems to speak against the above definition of shared intentional engagement, for apparently the meaning of the pain she refers to is not shared with others. But is this really so?

Let's first consider what language has to do with phenomenal experience. There is a sense in which one can say that only the person who has the sensation can know that she has an experience of pain: in theory, she could always pretend she is feeling pain. But can we deduce from the fact that only she is having that instance of a pain that the meaning of that pain sensation can be known only to her? I think that such a conclusion would be preposterous. Wittgenstein gives strong reasons against it in the context of his thoughts on the possibility of a “private language” in *Philosophical Investigations*. He admits that there is a sense in which somebody can attend to her experience that she could not describe to others—or herself (Wittgenstein, 1999, p. 277). But the impression one has at one moment is different from what is meant by sensation terms; the meaning of these terms needs to be recognized in repeated instances, which is done with the help of rules and criteria. Even if “pain” was only a word for something like “this feeling,” the deictic reference to “this feeling” would still be determined with the help of rules and criteria, which are at least potentially public. If there were no such criteria, the person having the pain herself would not know whether what she is having is a sensation, and less that it is a sensation of pain, rather than some other sensation. As a quality that can be recognized in other instances, the pain can be described to others and known to others.^2^

Wittgenstein's investigations into language match up well with the everyday experience of understanding other people's feelings. Of course, talking with somebody about her or his pain does not give us that person's pain. Since language and experience are different, there is always something about experience that cannot be conveyed by language. But speaking about somebody's pain can give us a pretty good idea of what the pain is like for the person. Our everyday experience is that of shared intentionality even when we refer to seemingly merely subjective feelings like pain. When doing so, we may make use of theory and simulation: we may theorize about the behavior of others, and we may try to relate it to sensations we know from our own experience. But the above consideration of the role of language for phenomenal experience suggests that phenomenal experience is not independent of rules and criteria that are expressed in language and pre-linguistic behavior. Because the rules and criteria of a language are shared between the speakers of the language, they enable shared intentional engagement.

The argument that experience is not independent of rules and criteria that are embedded in language and behavior is often misunderstood as the claim that language shapes in other ways unstructured experience. For instance, conventionalists claim that language carves out certain color experiences that could as well be carved out differently by different languages. Under this view, which hue in the (physical or phenomenal) color spectrum is called “blue” is conventional, and color words could just as well be assigned to different hues. I think, however, that this is not only a simplistic view of language, but that it also is inconsistent with the phenomenology of sensations. I now would like to shortly outline how phenomenological investigations can show that phenomenal experience itself is structured in many ways, and that these structures are not up to the individual subject.

There are some sensations that seem to force themselves upon us, or at least “pop out” from the stream of conscious experience. The experiential quality of a severe pain, for instance, demands attention, regardless of whether the pain has a serious cause or not. Other kinds of pain, such as a dull pain, are less prominent and sharply distinguished. In a similar way, a typical red, blue, or green seems to pop out much more than mixtures of these colors. In this sense, they have a characteristic phenomenal quality. For instance, when looking at a rainbow that has an equal distribution of wavelengths from infrared to ultraviolet, one would expect that the color gradient has a smooth appearance. But the phenomenal appearance of a rainbow is different; it looks as if some colors were more prominent than others, and as if there were steps in the distribution of colors. This may be the reason for why sensations are often thought to be self-intimating, that they reveal themselves to the person who has the experience just by having that experience. But this thought relies on the questionable assumption that individual phenomenal experiences are unaltered by such things as attention, the context of conscious experience, and learned distinctions, which would not only speak against the above considerations of language, but also is contradicted by the phenomenal structures of experience.

For instance, there is a structure to color sensations. One may imagine a subject that has inverted phenomenal experiences of yellow and blue, but such an inversion would at some point lead to different behaviors. When asked which experience looks brighter, the person with the inverted experiences would either have to answer that the blue looks brighter. Or, what she or he perceives as bright and dark would have to be inverted, too. Yet, due to the unequal distribution of hue, saturation, and brightness throughout the color spectrum, such inversions would become apparent with sufficient further intersubjective interaction.^3^ Studying the actual structure of color sensations shows that if “inverted qualia” are possible at all, then only to a very limited degree. Most phenomenal experiences cannot be completely different from one individual to another, and the relations between such qualitative experiences are not up to the individual. Because the structure of phenomenal experience is not something completely individual, it enables shared intentional engagement. This result of phenomenological study goes well together with the above remarks on language.

Philosophical investigations are often seen as at best relevant for meta-scientific considerations. But phenomenological discoveries such as that of pop out colors go well together with empirical research. For example, Berlin and Kay, in their famous study on basic color terms (1969) claim that, rather than picking out arbitrary parts of the color spectrum, basic color terms throughout a wide array of languages are clustered around foci. This suggests that there is something non-conventional about color terms, a suggestion that may receive further impetus by a study of the physiology of color perception. After all, the physiology of our sense organs and our nervous systems is relatively similar, in spite of important variances, which can sometimes lead to typical variations and aberrations. One way in which the build of the perceptual system could influence color vision is that human cone cells and neural structures react especially well to specific stimuli, which may cause the perception of focal colors. The phenomenal pop out experiences may, in turn, be the reason for why there are foci for basic color terms in a number of different languages. In a similar way, future empirical research into language and physiology may explain why there are shared structures in sense perception. An example of an interesting subject of further study in which phenomenology and empirical research can enrich each other are synesthetic experiences.

Even researchers who try to model basic color terms on a “purely cultural route” recognize that it is “driven, on its turn, by a non language-specific property of human beings,” which they take to be physiological (Loreto et al., 2012, p. 4). But, even though Loreto et al. proclaim a “non language-specific property” as the basis of color perception, they nevertheless model color terms as otherwise detached rather than part of a shared phenomenal structure. As with many authors who write on this topic, they imply the dichotomy I was arguing against above. On the one side, it is assumed that if language determines the right use of sensation terms, they are purely conventional. On the other side, it is presupposed that if there is a phenomenal quality to sensations, it is only contingently connected to language and behavior. If this were true, investigations of language and phenomenal qualities could never be brought together in a unified account of intersubjective interaction.

The idea that sensations are detached from behavior and language often goes back to what Fuchs and De Jaegher call the “‘inner world’ hypothesis.” They claim that it is presupposed by theory theory and simulation theory, both of which “conceive of the mental as an inner realm separated from others by an epistemic gulf that can only be crossed by inference or projection. We are hidden from each other in principle; therefore, we must infer or simulate the other's inner states in order to understand him” (Fuchs and De Jaegher, 2009, p. 467). But the above considerations of, on the one side, the role of shared language, and, on the other side, the shared phenomenal structure of experience, both suggest that we are not hidden from each other. Both show that already in repeatable phenomenal experience there is shared intentional engagement. We are thus not limited to theory and simulation when explaining other minds, although we may make use of both.

Because shared intentional engagement is fundamental for all kinds of human interaction, it is in need of interdisciplinary investigation, which has been hindered by the notion that the phenomenal experiences of humans are hidden from each other. Intentional engagement is conditioned by, amongst other things, language and its rules and criteria, forms of behavior, membership in cultures and social groups, the structure of phenomenal experience, the physiology of sense organs and neural structures, and much more. Scientific investigations into all of these can contribute to our understanding of how shared intentional engagement shapes intersubjective interaction. Investigations into intersubjective interactions thus need to integrate a number of diverse fields of research, such as psychology, psychiatry, neuroscience, and philosophy.

## Conflict of interest statement

The author declares that the research was conducted in the absence of any commercial or financial relationships that could be construed as a potential conflict of interest.

## Footnotes

^1^Examples of sensations are very different from the main examples of the debate around internalism and externalism that emanated from Putnam and Burge. The latter usually concern scientific concepts and not experiences, and involve something that is usually thought to be part of the external world, such as H_2_O.

^2^For further considerations of Wittgenstein's thoughts in this respect see Rudd (1999) and Durt (2014).

^3^Cf. e.g., Hilbert and Kalderon, 2000.
